# Trends in Influenza Infections in Three States of India from 2015–2021: Has There Been a Change during COVID-19 Pandemic?

**DOI:** 10.3390/tropicalmed7060110

**Published:** 2022-06-19

**Authors:** Anup Jayaram, Anitha Jagadesh, Ajay M. V. Kumar, Hayk Davtyan, Pruthu Thekkur, Victor J. Del Rio Vilas, Shrawan Kumar Mandal, Robin Sudandiradas, Naren Babu, Prasad Varamballi, Ujwal Shetty, Chiranjay Mukhopadhyay

**Affiliations:** 1 Manipal Institute of Virology, Manipal Academy of Higher Education, Manipal, Karnataka 576104, India; anitha.j@manipal.edu (A.J.); robin.s@manipal.edu (R.S.); naren.babu@manipal.edu (N.B.); prasad.varamballi@manipal.edu (P.V.); ujwal.shetty@manipal.edu (U.S.); chiranjay.m@manipal.edu (C.M.); 2 International Union against Tuberculosis and Lung Disease, 2 Rue Jean Lantier, 75001 Paris, France; akumar@theunion.org (A.M.V.K.); pruthu.tk@theunion.org (P.T.); 3 International Union against Tuberculosis and Lung Disease, South-East Asia Office, C-6 Qutub Institutional Area, New Delhi 110016, India; 4 Yenapoya Medical College, Yenapoya (Deemed to be University), University Road, Deralakatte, Manguluru 575018, India; 5 Tuberculosis Research and Prevention Centre NGO, Yerevan 0014, Armenia; info@tbrpc.am; 6 World Health Organization, World Health Emergencies, Southeast Asia Regional Office, New Delhi 110011, India; delriov@who.int; 7 Sukaraj Tropical and Infectious Disease Hospital, Teku, Kathmandu 44600, Nepal; fusion7722@gmail.com

**Keywords:** influenza, COVID-19, India, interrupted time series (ITS) analysis, influenza-like illness (ILI), severe acute respiratory illness (SARI), surveillance, SORT IT, operational research

## Abstract

The COVID-19 pandemic and public health response to the pandemic has caused huge setbacks in the management of other infectious diseases. In the present study, we aimed to (i) assess the trends in numbers of samples from patients with influenza-like illness and severe acute respiratory syndrome tested for influenza and the number and proportion of cases detected from 2015–2021 and (ii) examine if there were changes during the COVID-19 period (2020–2021) compared to the pre-COVID-19 period (2015–2019) in three states of India. The median (IQR) number of samples tested per month during the pre-COVID-19 period was 653 (395–1245), compared to 27 (11–98) during the COVID-19 period (*p* value < 0.001). The median (IQR) number of influenza cases detected per month during the pre-COVID-19 period was 190 (113–372), compared to 29 (27–30) during the COVID-19 period (*p* value < 0.001). Interrupted time series analysis (adjusting for seasonality and testing charges) confirmed a significant reduction in the total number of samples tested and influenza cases detected during the COVID-19 period. However, there was no change in the influenza positivity rate between pre-COVID-19 (29%) and COVID-19 (30%) period. These findings suggest that COVID-19-related disruptions, poor health-seeking behavior, and overburdened health systems might have led to a reduction in reported influenza cases rather than a true reduction in disease transmission.

## 1. Introduction

Influenza (or flu) is a contagious respiratory illness caused by influenza viruses and that spreads from person to person, mainly through airborne respiratory droplets generated from coughing and sneezing or direct contact with an infected surface or individual (skin-to-skin contact). It can cause illnesses that range in severity and sometimes lead to hospitalization and death—with the latter occurring mainly in high-risk groups, such as under-five children, the elderly, and people with immunosuppressive and chronic medical conditions [1]. According to the World Health Organization (WHO), seasonal influenza may infect up to 20% of the world’s population and results in 290,000–650,000 deaths every year [2,3]. There is a strong element of seasonality with outbreaks occurring mainly during the winter season in temperate climates, while in tropical regions, it may occur throughout the year. Because of annual outbreaks and occasional pandemics, the control of influenza has become a major public health challenge. According to data from the National Centre for Disease Control (NCDC), Government of India (GOI), India has reported 28,798 cases of Influenza A (H1N1) and 1218 deaths in 2019. Due to the ongoing COVID-19 pandemic, these numbers have drastically reduced to 2752 confirmed cases and 44 deaths in 2020 and 778 confirmed cases and 10 deaths in 2021 [4].

The COVID-19 disease is caused by the novel coronavirus SARS-CoV-2 and the COVID-19 pandemic has caused havoc throughout the globe. It has had a major impact on healthcare and social systems. Analysis of surveillance data from the United States, Australia, Chile, and South Africa show that there has been a significant decline in indicators of influenza activity, which include the number of samples submitted for influenza testing and the proportion of specimens testing positive [5]. This could be due to reduced health seeking for respiratory illness (due to lockdowns and mobility restriction measures) or interventions implemented to control the COVID-19 pandemic (such as closing of public places, mandatory use of face coverings, and social distancing), which might have had an impact on the incidence and the prevalence of influenza.

There is limited evidence on this issue from India. There is only one study from northern India which showed that there was a dramatic decline in influenza cases during 2020–2021 season (October to January) as compared to previous years [6]. With India being a country with vast geographic and climatic differences, there is a need to examine this issue in other parts of the country too. This will enable a clearer epidemiological picture at a regional/district level for better planning of preventive measures and interventions in case of future influenza outbreaks.

The aim of the study was to assess the trends in the numbers and proportions of samples tested for influenza, and the number of cases detected during the period of 2015–2021 and examine if there are changes in the trends during COVID-19 times (2020–2021) as compared to the pre-COVID-19 period (2015–2019) in three states of India. Specifically, we aimed to compare: (i) the average number of samples tested per month, ii) the number of influenza cases detected per month and their epidemiological distribution, and iii) the positivity rate for the influenza-like-illness (ILI) and severe acute respiratory infection (SARI) samples received from the Karnataka, Kerala, and Goa states.

## 2. Materials and Methods

### 2.1. Study Design

This was a cross-sectional study of routinely collected surveillance data.

### 2.2. Study Settings

#### General Setting: India

India is a country located in South Asia. It is the seventh-largest country by area and the second-most populous country [7]. It has twenty-nine states and nine union territories. India is home to an extraordinary variety of climatic regions, ranging from tropical in the south to temperate and alpine in the Himalayan north where elevated regions receive sustained winter snowfall. The health care system in India is a mixed one inclusive of public and private health-care service providers [8].

The study was conducted at Manipal Institute of Virology (MIV), a National Accreditation Board for Testing and Calibration Laboratories (NABL) accredited laboratory. MIV caters to the laboratory needs of three states: Karnataka, Kerala, and Goa. In 2004, a systematic laboratory-based surveillance network of influenza viruses was established by the Indian Council of Medical Research (ICMR), India. This network included nine clinical virology laboratories geographically distributed in Northern, Western, Eastern, Southern, and Central India [9]. Following the Influenza A(H1N1)pdm09 pandemic, NCDC, GOI developed an influenza surveillance laboratory work under an integrated disease surveillance program (IDSP) with the aim of strengthening and networking reference laboratories for prompt case confirmation and re-establishing the seasonal influenza surveillance system for India [10]. Under this network, there are currently 16 laboratories across India. These laboratories are part of the WHO Global Influenza Surveillance and Response System (GISRS), which serves as the global surveillance platform for seasonal, pandemic, and zoonotic influenza [11,12]. Since MIV works under a private–public partnership model and there is an excellent working relationship with state and district surveillance units, it receives samples from both private as well as government hospitals.

### 2.3. Study Population

Patients with ILI and SARI presenting in outpatient and inpatient departments of hospitals from three states, namely Karnataka, Kerala, and Goa, and whose samples were tested in MIV from 1 January to 31 December 2021 were included in the study.

### 2.4. Case Definitions

**ILI**: An acute respiratory infection with measured fever of ≥38 °C and cough with onset within the last 10 days [13].

**SARI**: An acute respiratory infection with measured fever of ≥38 °C and cough with onset within the last 10 days and requiring overnight hospitalization [13].

### 2.5. Clinical Data and Sample Collection and Transportation

Once patients are identified, their demographic and clinical details are recorded on a standardized-case-reporting form (CRF). Nasopharyngeal or nasal samples and throat swab samples are collected from each participant using an appropriate dacron swab and transported in viral transport medium to the laboratory in triple-layer packaging to maintaining the cold chain. Different modes of transportation are used to transport the samples. While hospitals from Kerala and Goa use the public train service to transport the samples, hospitals in Karnataka mostly use courier services barring those situated near MIV, which sends samples through patient attendees. A sample pick-up mechanism is developed by MIV to collect the samples coming by train. A laboratory staff goes to the railway station and collects the samples received in a parcel centre of the railway station at a specified time on a daily basis. All samples are delivered to MIV within 48 h after collection.

### 2.6. Testing at the Laboratory

Upon receipt of samples in the laboratory, they are checked if they fit the sample acceptance criteria, followed by sample registration and aliquoting of samples. The epidemiological and clinical information is entered into the laboratory information system.

Samples are processed within 24 h of receipt at the laboratory. Viral RNA is extracted using commercially available QIAamp Viral RNA Mini Kit (Qiagen GmbH, Hilden, Germany) as per manufacturer’s instructions. Amplification and detection for each RNA isolate is performed on Applied Biosystem’s Real Time PCR 7500/Quant Studio 5 instruments by using primers and probe sets for Influenza A and B as per the WHO real-time RT-PCR protocol.

Upon completion of tests, each result is verified by the virologist and approved by the laboratory manager before the data is entered into the laboratory information system and reported. The reports are sent by e-mail to the treating physician, district surveillance units, and state surveillance units with a copy to the central surveillance unit of IDSP. This mode of reporting helps with case management as well as with carrying out surveillance activity for public health action.

### 2.7. Analysis and Statistics

Data was downloaded from the electronic database and was analysed using Stata software version 16 (College Station, TX, USA: StataCorp LLC). The frequency and percentages were used to describe the socio-demographic and clinical characteristics of the samples tested for influenza during the pre-COVID-19 and COVID-19 periods. The influenza positivity rate (the percentage of samples positive for influenza out of the tested samples) was calculated for the pre-COVID-19 and COVID-19 periods. Time-trend graphs were used to depict the number of samples tested, the influenza cases detected, and the influenza positivity rates each month during the pre-COVID-19 and COVID-19 periods.

The number of samples tested (stratified by ILI and SARI) and the influenza cases detected per month during the study reference period (2015 to 2021) were deduced. The median and interquartile range (IQR) were used to summarize the monthly number of samples tested and number of influenza cases detected during the pre-COVID-19 and COVID-19 periods. The Mann–Whitney U test was used to assess the difference in the number of samples tested and influenza cases detected per month during the pre-COVID-19 and COVID-19 periods was statistically significant. The Chi-square test was used to assess the difference in the influenza positivity rates between the pre-COVID-19 and COVID-19 periods.

Interrupted time series (ITS) analysis was used to quantify the immediate and long-term effects of COVID-19 on the number of samples tested each month. January 2020, the month when the first case of COVID-19 was reported in India, was taken as an interruption in ITS analysis. We also conducted a sensitivity analysis using March 2020 as the interruption as most of the COVID-19 restrictions started at that time. Using the Newey–West regression methods, the predictive linear model for the pre-COVID-19 period (segment 1) with data from 60 months (January 2015 to December 2019) was developed to reflect the counterfactual during the COVID-19 period (January 2020 to December 2021). The predictive linear model accounted for secular trend, auto correlation, and seasonality. The beta coefficients (β) with 95% confidence interval (CI) were obtained for the intercept (starting point of the model) and the monthly trend (average increase or decrease in the numbers during consecutive months) in the number of samples tested during the pre-COVID-19 period. Similarly, the predictive linear model for the COVID-19 period (segment 2) with data from 24 months (January 2020 to December 2021) was developed, and the β coefficients for intercept and monthly trends were obtained.

The immediate effect of the COVID-19 interruption was quantified using level change in the intercept in the COVID-19 period compared to counterfactual for the interruption month obtained from the pre-COVID-19 period (expressed as β coefficients with 95% CI). A negative β coefficient for level change indicates immediate decline in the number of samples tested with the interruption. To assess the independent effect of COVID-19 on samples tested and influenza cases detected, an adjusted ITS analysis was conducted after adjusting for introduction of charges for testing from March 2018.

## 3. Results

**Figure 1:**
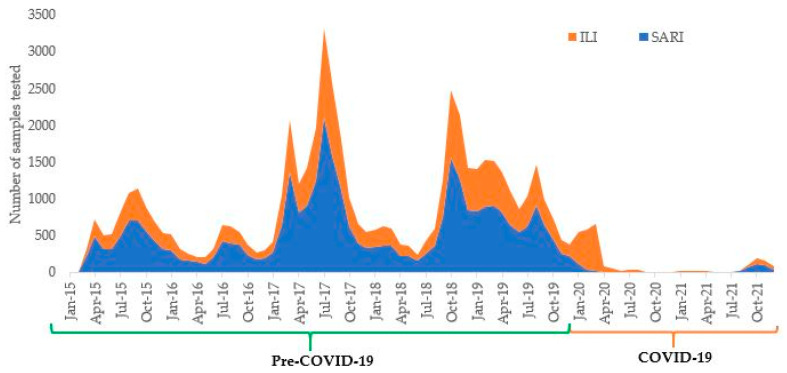
Clustered area diagram showing the number of samples tested (stratified by ILI and SARI) for influenza month-wise at MIV, Karnataka, India during 2015 to 2021. Abbreviations: MIV—Manipal Institute of Virology; ILI—influenza-like illness; SARI—severe acute respiratory illness.

In total, 54,262 samples were tested for influenza at MIV during the four years of the pre-COVID-19 period (2015–2019), and 2720 samples were tested over two years of the COVID-19 period (2020–2021). The median (IQR) number of samples tested per month during the pre-COVID-19 period was 653 (395–1245), compared to 27 (11–98) during the COVID-19 period (*p* value < 0.001). The median (IQR) number of ILI samples tested per month was 253 (155–493) and 21 (7–54) during the pre-COVID-19 and COVID-19 periods, respectively (*p* value < 0.001). Similarly, the median (IQR) number of SARI samples tested per month during the pre-COVID-19 period was 415 (246–795) as compared to 8 (0–29) during the COVID-19 period (*p* value < 0.001) (Table 1). Figure 1 depicts the trend in the number of samples tested each month and shows a clear decline in the COVID-19 period compared to the pre-COVID-19 period.

Of the samples tested, the majority were from females and came from the Karnataka state during the pre-COVID-19 and COVID-19 periods. About 20% of the samples during the pre-COVID-19 period were from pediatric patients (aged < 15 years) compared to 11% during the COVID-19 period. During the pre-COVID-19 period, the majority of the samples were from SARI patients (62%) in contrast to ILI patients (79%) during the COVID-19 period (Table 2).

**Figure 2:**
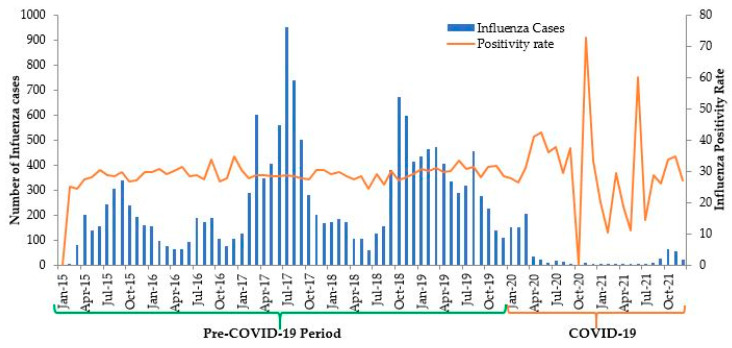
Line diagram depicting the number of influenza cases detected month-wise and test positivity rate at MIV, Karnataka, India during 2015 to 2021. Abbreviations: MIV—Manipal Institute of Virology; ILI—influenza-like illness; SARI—severe acute respiratory illness.

In total, 15,752 and 812 influenza cases were detected during the pre-COVID-19 period and COVID-19 periods, respectively. The median (IQR) number of influenza cases detected per month during the pre-COVID-19 period was 190 (113–372), compared to 29 (27–30) during the COVID-19 period (*p* value < 0.001). However, there was no change in the overall influenza positivity rate between the pre-COVID-19 (29%) and COVID-19 (30%) periods—this lack of difference persisted across patient sub-groups (Table 2). Figure 2 depicts the decline in the number of influenza cases detected each month in the COVID-19 period compared to the pre-COVID-19 period. Of the influenza cases detected, the majority were Influenza A (H1N1)pdm09 during both the pre-COVID-19 (59%) and COVID-19 (58%) periods. There was no statistically significant variation in the pattern of strains of influenza cases detected during the pre-COVID-19 and COVID-19 periods (Table 1).

**Figure 3:**
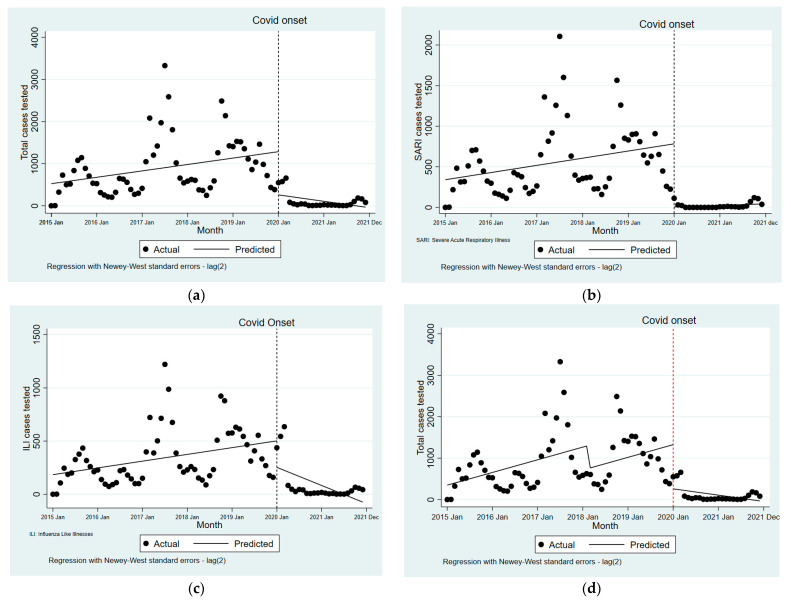
Interrupted time series showing the unadjusted and adjusted linear trend in the number of samples tested for influenza (stratified by ILI and SARI) each month at MIV, Karnataka, India during Pre-COVID-19 (segment-1) and COVID-19 periods (segment-2). (**a**) Total samples tested; (**b**) SARI cases tested; (**c**) ILI cases tested; (**d**) total samples tested adjusted for charges for testing; Segment-1: pre-COVID-19 (2015–2019), Segment-2: COVID-19 period (2020–2021); interruption: month the first of case of COVID-19 was reported (January 2020). Abbreviation: MIV—Manipal Institute of Virology.

Interrupted time series (ITS) analysis showed a significant reduction in the total number of samples tested (β coefficient = −1025.6, 95% CI: −1588.5 to −462.6) during January 2020 (COVID-19 interruption) compared to that without COVID-19 (counterfactual from pre-COVID-19 period). The change in the total number of samples tested was significantly negative (β coefficient = −1067.1, 95% CI: −1657.2 to −477.0) even after adjusting for the introduction of charges for testing. In the adjusted analysis, there was a significant reduction in the SARI samples tested (β coefficient = −804.6, 95% CI: −1116.8 to −492.4) during January 2020 but not in ILI samples tested (β coefficient = −262.5, 95% CI: −582.0 to 57.0). With the adjusted analysis, there was a significant declining trend in the total number of samples tested (β coefficient = −38.2, 95% CI: −69.5 to −6.9) during the COVID-19 period (Figure 3 and Table 3).

**Figure 4:**
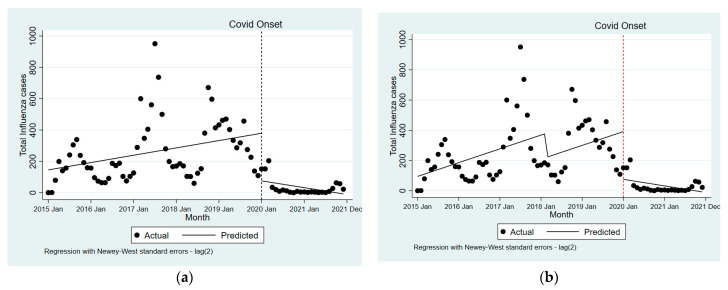
Interrupted time series showing the unadjusted and adjusted linear trend in the number of influenza cases detected each month at MIV, Karnataka, India during pre-COVID-19 (segment-1) and COVID-19 periods (segment-2). (**a**) Total influenza cases detected; (**b**) total influenza cases detected adjusted for charges for testing; Segment-1: pre-COVID-19 (2015–2019), Segment-2: COVID-19 period (2020–2021); interruption: month the first of case of COVID was reported (January 2020). Abbreviation: MIV—Manipal Institute of Virology.

Adjusted ITS analysis showed a significant reduction in the total number of influenza cases detected (β coefficient = −316.1, 95% CI: −483.3 to −149.0) during January 2020 compared to that without COVID-19. There was a significant declining trend in the number of influenza cases detected (β coefficient = −11.2, 95% CI: −20.1 to −2.3) during the COVID-19 period (Figure 4 and Table 3).

In the sensitivity analysis taking March 2020 as the cut-off for the onset of the COVID-19 pandemic, it was seen that the reduction in the number of samples tested (both for ILI and SARI) and influenza cases detected was significant and steeper compared to the analysis presented above with January 2020 as the interruption month (Supplementary File S1).

## 4. Discussion

This is the first study from south India to assess the impact of the COVID-19 pandemic on the trends in influenza case detection. We had four key findings. First, there was a drastic reduction in the number of ILI and SARI samples tested for influenza during the COVID-19 period compared to the pre-COVID-19 period. The decline was relatively steeper for SARI samples tested compared to ILI samples. The impact of COVID-19 remained even after adjusting for introduction of charges, thus demonstrating the independent impact of COVID-19 on influenza trends. Second, there was a similar drastic reduction in the number of influenza cases detected. Third, the declining trend of both samples tested and influenza cases detected continued for most of the COVID-19 period with some recovery towards the end of 2021. Fourth, the influenza positivity rate and the distribution of the influenza sub-types remained unchanged during the COVID-19 period compared to the pre-COVID-19 period.

The reduction in influenza cases detected during COVID-19 times might be due to many reasons, which include (i) reduced number of people with ILI/SARI visiting the health facilities as a result of mobility restrictions and lockdowns, (ii) reduced number of samples reaching the laboratory due to change in surveillance protocols, or (iii) reduced transmission of influenza virus due to implementation of non-pharmacological interventions to curb COVID-19, such as prohibition of public gatherings, closing of schools and workplaces, social distancing, use of masks, and improved hand hygiene. There is sound evidence from China, Hong Kong, USA, Singapore, and Australia that there was indeed a reduction in transmission of influenza and other respiratory diseases during the COVID-19 times [5,14,15,16].

We assume that the reduction of influenza cases in our setting was not due to a reduction in influenza transmission for the following reasons. First, the lockdowns and mobility restrictions were severe during the initial part of the COVID-19 pandemic, which might have reduced the number of footfalls to the health facilities nationwide. Second, there was indeed a change in protocol during the COVID-19 times. ILI/SARI patients visiting the health facilities were prioritized for COVID-19 testing and only samples tested negative for COVID-19 were transported for influenza testing. Third, there was no change in the influenza positivity rate in our study in contrast to studies from USA, Singapore, and Australia [5,14] where there was a reduction of not only the number of influenza cases but also a reduction in the influenza positivity rate. Influenza positivity is an important indicator of influenza transmission. Since there was no change in this indicator in our study, we are of the opinion that the reduction in influenza cases detected was less likely due to a reduction in transmission.

There were several strengths to this study. First, we used the surveillance data from an influenza surveillance laboratory which catered to three states of southern India. Thus, the data generated can be considered representative of the epidemiological picture of influenza in these three states. Second, we performed a robust ITS analysis with data points spanning seven years (five years for the pre-COVID-19 period and two years for the COVID-19 period), thus accounting and adjusting for seasonal changes that might have affected influenza case detection. Third, the conduct and reporting of the study were in line with the Strengthening the Reporting of Observational Studies in Epidemiology (STROBE) guidelines [17].

However, there were some limitations too. Our study was limited to three states of India; hence, it may not be nationally representative. With India being a large country with varied climatic conditions and ecology, the findings of this study may not be same for other regions of the country. We did not have information on the number of ILI/SARI patients who visited the health facilities and in what proportion of these were the samples sent for influenza testing. Such indicators would have added value to our analysis. Finally, our study was limited to analysis of quantitative data received by the surveillance laboratory. Qualitative exploration using interviews and focus group discussions of patients and providers would have added value to explain the quantitative findings. These points will be explored in future research studies.

Despite these limitations, there are some important programmatic implications from this study. First, the surveillance system needs to be expanded to include important additional indicators, such as (i) number of patients with ILI/SARI visiting the health facilities, (ii) number of ILI/SARI samples collected and transported, and (iii) influenza positivity rate. This will help in a comprehensive analysis of surveillance data and track influenza transmission. Second, the number of samples reaching the laboratory remained low during most of the COVID-19 period with some semblance of recovery only towards the end of 2021. A vigilant surveillance system would have acted upon the findings early in the epidemic and would have instituted corrective measures to mitigate the impact. This needs to be taken note of by all the stakeholders involved in the surveillance system. As a mitigation strategy, the operational guidelines on influenza surveillance have to clearly describe the actions to be taken to negate the disruption due to pandemics in the future. Also, sentinel surveillance sites can deduce a site-specific plan for continued service during any pandemic. Finally, analyses such as that undertaken in this study should be undertaken at periodic intervals to track the change going forward so that appropriate actions can be initiated quickly.

## 5. Conclusions

Using the existing influenza surveillance platform in three states of India, we documented the number ILI/SARI samples tested and the number and proportion of influenza cases detected in relation to the COVID-19 pandemic. We found that there was a dramatic reduction in the number of samples tested and the number of influenza cases detected during the COVID-19 period compared to the pre-COVID-19 period. However, there was no change in the positivity rate, possibly indicating that there was no reduction in influenza transmission. We recommend that the influenza surveillance system be strengthened by inclusion of additional indicators and real-time analysis leading to corrective action.

## Figures and Tables

**Table 1:** Number of samples tested for influenza and pattern of influenza cases detected at MIV, Karnataka during the pre-COVID-19 (2015 to 2019) and COVID-19 periods (2020 to 2021).

Parameters	Pre-COVID-19 (2015 to 2019)		COVID-19 (2020 to 2021)		*p* Value
*Testing*					
Number of samples tested (a)	54,262		2720		
Median (IQR) number of samples tested per month [all samples]	653	(395-1245)	27	(11–98)	<0.001 *
ILI	253	(155-493)	21	(7–54)	<0.001 *
SARI	415	(246-795)	8	(0–29)	<0.001 *
*Influenza cases detected*					
Number of influenza cases detected (b)	15,752		812		
Median (IQR) number of influenza cases detected per month	190	(113-372)	29	(27–30)	<0.001 *
Overall positivity rate (b*100/a)	29.0%		29.9%		0.356
*Pattern of influenza*					
Number (%) ^$^ of A (H1N1) pdm09	9359	(59.4)	473	(58.3)	0.510 ^#^
Number (%) ^$^ of A/H3N2	3485	(22.1)	174	(21.4)	0.641 ^#^
Number (%) ^$^ of Influenza B	2908	(18.5)	165	(20.3)	0.184 ^#^

* Mann–Whitney U test; ^#^ Chi-square test; ^$^ percentage calculated with total number of influenza cases detected in pre-COVID-19 (15,752) and COVID-19 (812) periods as denominator.

**Table 2:** Socio-demographic and clinical characteristics of the patients who were tested for influenza and test positivity rate at MIV, Karnataka during the pre-COVID-19 (2015 to 2019) and COVID-19 periods (2020 to 2021).

Characteristics	Pre-COVID-19 (2015–2019)				COVID-19 (2020–2021)				*p* Value ^$^
	Tested		Positive		Tested		Positive		
	n	(%) *	n	(%) ^#^	n	(%) *	n	(%) ^#^	
***Total***	54,262	(100)	15752	(29.0)	2720	(100)	812	(29.9)	0.356
**Age in years**									
<5	6925	(12.8)	1844	(26.6)	168	(6.2)	39	(23.2)	0.322
5-14	3989	(7.4)	1374	(34.4)	125	(4.6)	43	(34.4)	0.992
15-24	6323	(11.7)	1760	(27.8)	508	(18.7)	159	(31.3)	0.095
25-34	8191	(15.1)	1937	(23.7)	470	(17.3)	101	(21.5)	0.283
35-44	6040	(11.1)	1996	(33.1)	269	(9.9)	86	(32.0)	0.713
45-54	6602	(12.2)	1814	(27.5)	260	(9.6)	63	(24.2)	0.249
55-64	7023	(12.9)	1824	(26.0)	361	(13.3)	110	(30.5)	0.058
>65	9169	(16.9)	3203	(34.9)	559	(20.6)	211	(37.8)	0.176
**Gender**									
Male	25,784	(47.5)	7175	(27.8)	1303	(47.9)	375	(28.8)	0.454
Female	28,478	(52.5)	8577	(30.1)	1417	(52.1)	437	(30.8)	0.563
**State**									
Karnataka	31,905	(58.8)	9187	(28.8)	1647	(60.6)	486	(29.5)	0.533
Goa	3472	(6.4)	974	(28.1)	124	(4.6)	30	(24.2)	0.347
Kerala	17,332	(31.9)	5111	(29.5)	877	(32.2)	269	(30.7)	0.453
Others	1553	(2.9)	480	(30.9)	72	(2.6)	27	(37.5)	0.238
**Clinical Case**									
ILI	20,545	(37.9)	5837	(28.4)	2145	(78.9)	639	(29.8)	0.178
SARI	33,717	(62.1)	9915	(29.4)	575	(21.1)	173	(30.1)	0.723
**Type of Sample**									
Diagnosis	43,093	(79.4)	12340	(28.6)	2220	(81.6)	669	(30.1)	0.128
Surveillance	11,169	(20.6)	3412	(30.6)	500	(18.4)	143	(28.6)	0.354

* Row percentage; ^#^ column percentage; ^$^ Chi-square test comparing the positivity rate in each of the rows in the table. Abbreviations: ILI—influenza-like illness; SARI—severe acute respiratory illness.

**Table 3:** Interrupted time series analysis of monthly number of samples tested for influenza and influenza cases detected at MIV, Karnataka, India during pre-COVID-19 period (2015–2019) and COVID-19 period (2020–2021).

Particulars	Pre-COVID-19 Trend (Segment-1)		LVC Versus without COVID-19		COVID-19 Trend (Segment-2)	
*Unadjusted*						
Total samples tested	12.7	(2.3 to 23.1)	−1025.6	(−1588.5 to −462.6)	−25.3	(−45.3 to −5.4)
ILI samples tested	5.3	(1.2 to 9.4)	−248.9	(−561.2 to 63.4)	−19.5	(−34.8 to −4.2)
SARI samples tested	7.4	(1.0 to 13.7)	−776.7	(−1068.3 to −485.0)	−5.8	(−12.6 to 1.0)
Influenza cases detected	3.9	(0.9 to 6.9)	−304.2	(−463.7 to −144.6)	−7.5	(−13.2 to −1.8)
Adjusted *						
Total samples tested	25.5	(−0.9 to 51.9)	−1067.1	(−1657.2 to −477.0)	−38.2	(−69.5 to −6.9)
ILI samples tested	9.5	(−0.2 to 19.2)	−262.5	(−582.0 to 57.0)	−23.7	(−41.4 to −6.0)
SARI samples tested	16.0	(−0.7 to 32.8)	−804.6	(−1116.8 to −492.4)	−14.5	(−31.3 to 2.4)
Influenza cases detected	7.6	(0.1 to 15.1)	−316.1	(−483.3 to −149.0)	−11.2	(−20.1 to −2.3)

Note: Data are beta (β) coefficients with 95% CI in the brackets from the linear regression model *; both unadjusted and adjusted models accounted for the secular trend, auto correlation, seasonality; * the model was adjusted for introduction of charges for tests (introduced in March 2018); Segment-1: pre-COVID-19 period (2015–2019); Segment-2: COVID-19 period (2020–2021); interruption: month the first of case of COVID was reported. Abbreviations: LVC—level change in January 2020; ILI—influenza-like illness; SARI—severe acute respiratory illness.

## Data Availability

The data that support the findings of the study are available from the corresponding author (A.J. (Anup Jayaram)) upon reasonable request.

## Supplementary Materials

The following supporting information can be downloaded at: https://www.mdpi.com/article/10.3390/tropicalmed7060110/s1, Figure S1: Interrupted time series showing the unadjusted and adjusted linear trends in the number of samples tested for influenza (stratified by ILI and SARI) each month at MIV, Karnataka, India during the pre-COVID (segment-1) and COVID periods (segment-2); Table S1: Interrupted time series analysis of monthly number of samples tested for influenza and influenza cases detected at MIV, Karnataka, India during the pre-COVID period (January 2015 to February 2020) and the COVID period (March 2020 to December 2021); Figure S2: Interrupted time series showing the unadjusted and adjusted linear trend in the number of influenza cases detected each month at MIV, Karnataka, India during the pre-COVID (segment-1) and COVID periods (segment-2).
