# Supplementary material for: Trends in Influenza Infections in Three States of India from 2015–2021: Has There Been a Change during COVID-19 Pandemic?
Source: Trop Med Infect Dis. 2022 Jun 19;7(6):110. doi: 10.3390/tropicalmed7060110 (PMC9228248; doi:10.3390/tropicalmed7060110)
Supplement: Supplementary file 1 [file tropicalmed-07-00110-s001.zip › tropicalmed-1742460-supplementary.pdf]

**Table S1:** Interrupted time series analysis of monthly number of samples tested for influenza and influenza cases detected at MIV, Karnataka, India during Pre-COVID period (January 2015 to February 2020) and COVID period (March 2020 to December 2021)

| Particulars | Pre-COVID trend (Segment-1) | | LVC versus without COVID | | COVID trend (Segment-2) | | Overall trend (Pre-COVID and COVID) | |
|---|---|---|---|---|---|---|---|---|
| *Unadjusted* | | | | | | | | |
| Total samples tested | 10.5 | (0.1 to 20.8) | -1099.3 | (-1628.5 to -570.1) | -14.8 | (-31.1 to 1.6) | -4.3 | (-18.8 to 10.3) |
| ILI samples tested | 5.3 | (1.6 to 8.9) | -377.4 | (-619.5 to -135.4) | -12.9 | (-26.1 to 0.3) | -7.6 | (-20.3 to 5) |
| SARI samples tested | 5.2 | (-1.9 to 12.3) | -721.8 | (-1035.2 to -408.5) | -1.9 | (-9.2 to 5.5) | 3.3 | (0.5 to 6.2) |
| Influenza cases detected | 3.2 | (0.2 to 6.2) | -319.4 | (-476.5 to -162.2) | -4.7 | (-9.8 to 0.4) | -1.5 | (-6.1 to 3.1) |
| *Adjusted** | | | | | | | | |
| Total samples tested | 22.5 | (-3.3 to 48.4) | -1157.3 | (-1729.2 to -585.4) | -26.8 | (-55.3 to 1.7) | -4.3 | (-18.9 to 10.3) |
| ILI samples tested | 9.4 | (0.1 to 18.7) | -397.3 | (-650 to -144.6) | -17 | (-32.8 to -1.3) | -7.6 | (-20.4 to 5.1) |
| SARI samples tested | 13.1 | (-3.7 to 29.9) | -760 | (-1104.9 to -415.1) | -9.8 | (-26.7 to 7.1) | 3.3 | (0.4 to 6.3) |
| Influenza cases detected | 6.7 | (-0.7 to 14) | -336 | (-505 to -167) | -8.2 | (-16.4 to 0.1) | -1.5 | (-6.1 to 3.2) |

Note: Data are Beta (β) Coefficients with 95% CI in the brackets from the linear regression model*; Both unadjusted and adjusted models accounted for the secular trend, auto correlation, seasonality; *The model was adjusted for introduction of charges for tests (introduced in March 2018); Segment-1: Pre-COVID Period (2015-2019); Segment-2: COVID Period (2020-2021); Interruption: March 2020- Month the first of case of COVID without history of international travel and institution of lockdown. Abbreviations: LVC- Level change in March 2020; ILI- Influenza like Illness; SARI- Severe Acute Respiratory Illness

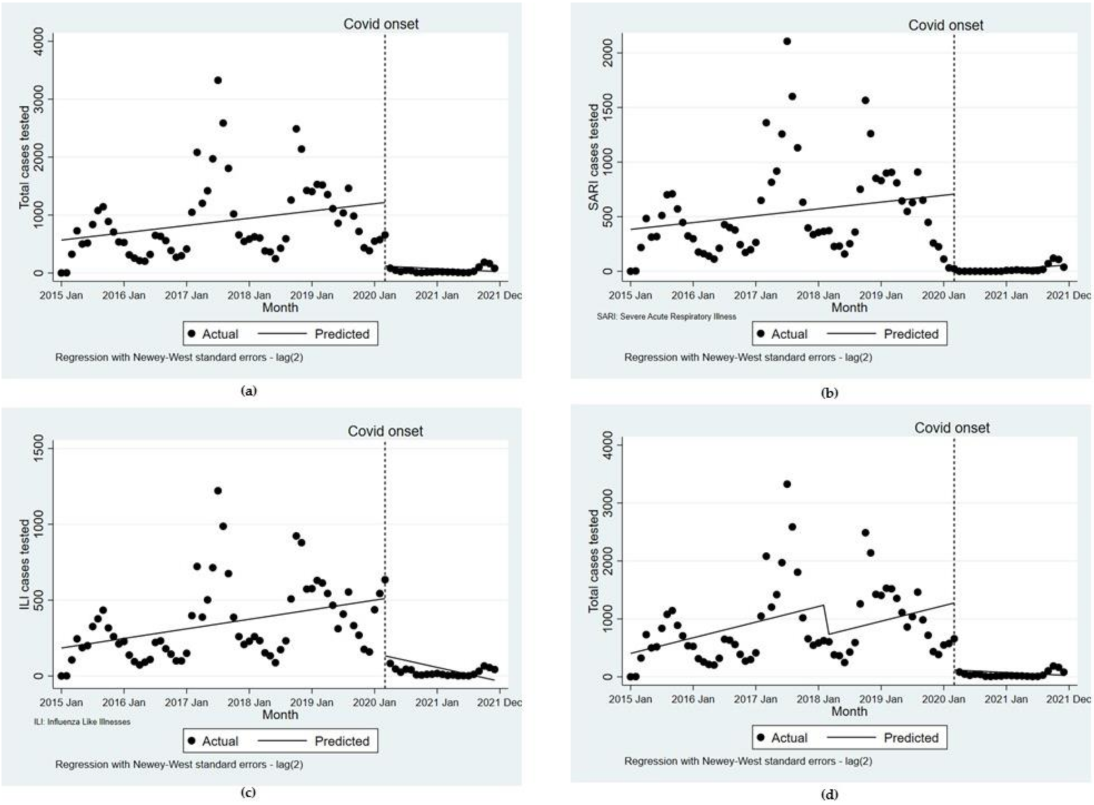


**Figure S1:** Interrupted time series showing the unadjusted and adjusted linear trend in the number of samples tested for Influenza (stratified by ILI and SARI) each month at MIV, Karnataka, India during Pre-COVID (segment-1) and COVID period (segment-2).

(a) Total samples tested; (b) SARI cases tested; (c) ILI cases tested; (d) Total samples tested adjusted for charges for testing; Segment-1: Pre-COVID (January 2015 to February 2020), Segment-2: COVID Period (March 2020 to December 2021); Interruption: Month the first of case of COVID was reported (March 2020). Abbreviation: MIV: Manipal Institute of Virology

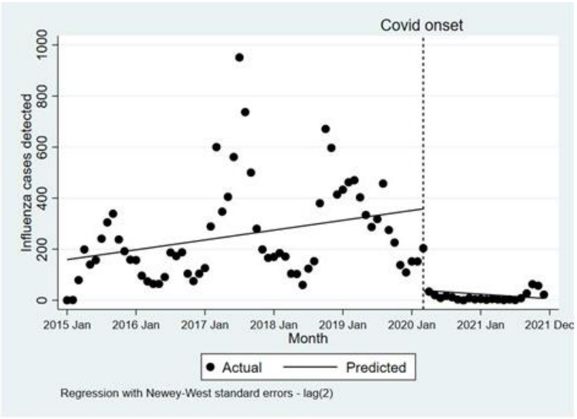


(a)

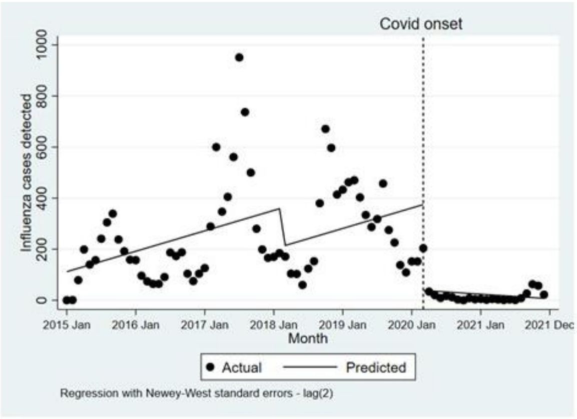


(b)

**Figure S2:** Interrupted time series showing the unadjusted and adjusted linear trend in the number of Influenza cases detected each month at MIV, Karnataka, India during Pre-COVID (segment-1) and COVID period (segment-2).

(a) Total influenza cases detected; (b) Total influenza cases detected adjusted for charges for testing; Segment-1: Pre-COVID (January 2015 to February 2020), Segment-2: COVID Period (March 2020 to December 2021); Interruption: Month the first of case of COVID was reported (March 2020). Abbreviation: MIV: Manipal Institute of Virology
